# The NAS Perchlorate Review: Ginsberg et al. Respond

**Published:** 2005-11

**Authors:** Gary Ginsberg, Deborah Rice

**Affiliations:** Connecticut Department of Public Health, Hartford, Connecticut, E-mail: gary.ginsberg@po.state.ct.us; Maine Bureau of Health, Augusta, Maine

We would like to respond to the comments from several members of the NAS perchlorate panel (Johnston et al.) and from two other groups (Gibbs et al., Strawson et al.). These letters were in response to our commentary published in *EHP* (Ginsberg and Rice 2005). The letters take an opposing viewpoint but do not invalidate our main assertions that *a*) the low dose reported in the Greer et al. study (Greer et al. 2002) does in fact demonstrate a majority of subjects with the perchlorate-induced effect; *b*) there is the potential for greater perchlorate vulnerability in pregnant women and newborns than in the general population; and *c*) inhibition of iodide uptake is a key step in the perchlorate toxicodynamic pathway, with moderate levels of uptake inhibition potentially sufficient to produce adverse effects in sensitive subgroups.

The low dose reported by Greer et al. (2002) was termed a no observable effect level (NOEL) by the National Research Council (NRC 2005). We disagreed with this view in our commentary because four of seven individuals at this dose showed the characteristic perchlorate-induced suppression of iodine uptake. Johnston et al. claim that we ignored the nonresponders when we described the low dose in the Greer study (Greer et al. 2002) as an effect level. We did not disregard these subjects, but we pointed out that they segregate out as a subgroup who appear to be less sensitive to the perchlorate effect and have low baseline values. We further pointed out that, because of the small sample size (*n* = 7), there is very little statistical power to detect an effect at Greer et al.’s low dose (0.007 mg/kg/day) given the variability in response. Rather than simply relying on a weak test of significance, our closer inspection of the data indicated that the majority of the low-dose subjects were responders. When the results are organized categorically into responders and nonresponders, it is evident that the low dose is part of the dose–response continuum with no evidence of a threshold: 0.5 mg/kg/day, 9 responders out of 9 subjects; 0.1 mg/kg/day, 10 responders out of 10 subjects; 0.02 mg/kg/day, 6 responders out of 10 subjects; and 0.007 mg/kg/day, 4 responders out of 7 subjects. The lack of statistical significance should not be used as grounds for disqualifying what appears to be a biologically significant response.

Hydrogen sulfide provides a good example for illustrating biologic versus statistical significance. In a key study, Jappinen et al. (1990) found that an inhaled dose of hydrogen sulfide did not cause a statistically significant effect on airway parameters in a group of 10 subjects with asthma. However, when these data were used by the Agency for Toxic Substances and Disease Registry (ATSDR) to set a public health benchmark (the acute minimum risk level), the fact that 2 of the 10 asthmatics were responders was sufficient for this dose to be considered a critical effect level (ATSDR 1999). The perchlorate low-dose responders should not be ignored, just as the hydrogen sulfide low-dose responders were not ignored.

Although the NRC considered Greer et al.’s (2002) low dose a NOEL, like us, the U.S. Environmental Protection Agency (EPA) draft assessment (U.S. EPA 2002) and a risk assessment by the Massachusetts Department of Environmental Protection (Mass DEP 2004) considered this dose to be a LOAEL (lowest observed adverse effect level). The California EPA conducted a benchmark dose analysis on the data published by Greer et al. (2002), finding 0.0037 mg/kg/day (approximately 2-fold below Greer et al.’s low dose) the critical point of departure for standard setting (California EPA 2004).

Strawson et al. make the argument that the critical adverse effect of perchlorate is hypothyroidism. It is important to understand that clinical hypothyroidism is not the critical end point for derivation of the perchlorate RfD. Subclinical hypothyroidism in pregnant women can result in adverse nervous system effects in offspring (Zoeller et al. 2002), including decreased IQ (Haddow et al. 1999). Perchlorate’s inhibition of iodine uptake increases the risk for hypothyroidism, which even if subclinical, may still be associated with neurodevelopmental effects.

The rebuttal letters (Gibbs et al., Johnston et al., and Strawson et al.) consider inhibition of iodine uptake a nonadverse effect because it is only temporary and because compensatory homeostatic mechanisms would not allow actual declines in thyroid hormone to occur. They cite an abstract by Braverman et al. (2004) to demonstrate that the perchlorate effects seen by Greer et al. (2002) disappear upon longer-term (6 month) exposure. As we pointed out in our commentary (Ginsberg and Rice 2005), the study by Braverman et al. (2004) has not been published or peer reviewed and involves small numbers of subjects. It is unclear whether there was sufficient statistical power to see the perchlorate effect. Since the publication of our commentary we became aware of a different study by this same group (Braverman et al. 2005). Gibbs et al. also mentioned this study. In contrast to their abstract (Braverman et al. 2004), Braverman et al. (2005) show iodine uptake inhibition in relatively young male Caucasian workers who had a median perchlorate exposure period of 5.9 years. The dose response for these long-term perchlorate workers was similar to that shown for subjects exposed to perchlorate for 2 weeks (Greer et al. 2002). This suggests that, contrary to the NRC report (NRC 2005) and Braverman et al. (2004), perchlorate does not lose its potency to inhibit iodide uptake under conditions of long-term exposure.

The fact that the workers in the study by Braverman et al. (2005) did not have indications of thyroid deficiency suggests that healthy workers can compensate for this type of biochemical impairment. This is likely due to several factors, including sufficient iodide and hormone reserves in these workers. However, it is uncertain that perchlorate-induced impairment of iodine uptake would be compensated for in all members of the population. In particular, a substantial percentage of the general public has low iodine intake [Centers for Disease Control and Prevention (CDC) 2000; Hollowell et al. 1998], pregnant women can be at greater risk for iodine deficiency (Azizi et al. 2003), and the neonate appears to have minimal stores of thyroid hormone (Delange 1998; van den Hove et al. 1999). In addition, the data of Braverman et al. (2005) suggest up-regulation of the iodide symporter in these workers, a protective mechanism that may not exist in the fetus or neonate. Infants have added susceptibility because perchlorate is excreted into breast milk and appears to inhibit iodine secretion into breast milk (Kirk et al. 2005).

On this last point, the letter by Gibbs et al. casts doubt on the relationship between perchlorate and iodine levels in breast milk by quoting from Kirk et al. (2005): “If we take all the available data, there is no meaningful correlation between the perchlorate and iodide levels in breast milk.” This is a case of selective quoting, as the very next sentence states, “On the other hand, for breast milk that contained ≥10 μg/L perchlorate, the iodide concentration expressed in milk is linearly related to the reciprocal of perchlorate concentration.” Although we would agree that the findings of Kirk et al. (2005) need to be further explored, Gibbs et al.’s dismissal of these findings—on the basis of an out-of-context quote—is misleading.

Strawson et al. claim in their letter that the NRC used a nonstandard approach in deriving the perchlorate RfD. Citing an article by Barnes and Dourson (1988), they state that there are two possible approaches to developing an RfD: the use of a NOAEL of a critical effect from an adult population, or the use of the NOAEL of a precursor effect in a sensitive population. Barnes and Dourson (1988) did not discuss such a dichotomy of approaches, nor did more recent U.S. EPA guidance (e.g., U.S. EPA 1991, 2002). In fact, IRIS (the Intergrated Risk Information System; IRIS 2005) defines “critical effect” as “[T]he first adverse effect, or its known precursor, that occurs to the most sensitive species.” There is no distinction in any of these documents made for critical end point being chosen based on sensitive population, nor is there discussion of “immediate precursor” versus other precursors, a distinction made by Strawson et al. Therefore, the assertion that the NRC used a nonstandard approach in using a precursor event in a nonsensitive population (adults) is not supported in U.S. EPA guidance (U.S. EPA 1991, 2002).

Also at issue are the uncertainty factors that need to be applied to the data of Greer et al. (2002) to derive a health-protective RfD. The NRC risk assessment included a total 10-fold uncertainty factor (NRC 2005). This factor is expected to cover a lot of ground: variability in toxicokinetics and toxicodynamics among healthy adults, variability caused by low iodine uptake, pregnancy, neonatal vulnerabilities described above, and the data gaps and temporal uncertainties described in our commentary (Ginsberg and Rice 2005). Because of these factors, our scientific judgment is that a 10-fold uncertainty factor is insufficient, which is the same judgment arrived at in the U.S. EPA draft assessment (U.S. EPA 2002) and in the Massachusetts risk assessment (Mass DEP 2004).

The letters of Gibbs et al. and Strawson et al. allude to the Chilean data set (Crump et al. 2000; Tellez et al. 2005) as documenting that early life stages are not especially affected by relatively high exposure to perchlorate in drinking water. If this were the case, it would decrease the level of uncertainty contained in the risk assessment. However, in our commentary (Ginsberg and Rice 2005), we pointed out the limitations of the Chilean data. It requires extrapolation from an iodine-enriched population in Chile to the United States, which has considerably less iodine intake. Further, nearly 5% of school-age children and 15% of women of childbearing age in the United States have low iodine intake (CDC 2000; Hollowell et al. 1998) these individuals are likely not well represented by the Chilean data set. Crump et al. (2000) show an association between high perchlorate in drinking water and family history of thyroid disease. The fact that this association did not extend to altered thyroid status in the children studied raises the possibility that iodine supplementation efforts in recent decades in Chile prevented the perchlorate effect in current-day children (Crump et al. 2000). This leaves open the question of perchlorate-induced effects in children in the United States whose iodine intake is suboptimal. A follow-up study by Tellez et al. (2005) reproduces some of the earlier Chilean findings but shows that in spite of very recent reductions in the iodide content of salt in Chile, iodine levels are still approximately 2-fold higher there than in the United States. The Chilean studies do not remove the uncertainties present in the perchlorate database.

Our disagreement with the NAS perchlorate document (NRC 2005) and with these letters centers around how a no effect level is defined and how vulnerable life stages are factored into a risk assessment. These authors recommend stretching the definition of NOEL to include a dose level in which the majority of the subjects demonstrate the perchlorate effect. Gibbs et al., Johnston et al., and Strawson et al. also recommend using studies of healthy adults and a poorly matched Chilean population to dismiss the adverse nature of perchlorate-induced iodide uptake inhibition for vulnerable subgroups. As state risk assessors, we strive to keep methods and judgment consistent across all chemicals. Applying that to perchlorate leads us to a different analysis than what was presented by the NAS and what is promoted in the letters responding to our commentary (Ginsberg and Rice 2005).
